# Consumers’ Perceptions of Five Front-of-Package Nutrition Labels: An Experimental Study Across 12 Countries

**DOI:** 10.3390/nu11081934

**Published:** 2019-08-16

**Authors:** Zenobia Talati, Manon Egnell, Serge Hercberg, Chantal Julia, Simone Pettigrew

**Affiliations:** 1 School of Psychology, Curtin University, Kent St, Bentley, WA 6102, Australia; 2 Nutritional Epidemiology Research Team (EREN), Sorbonne Paris Cité Epidemiology and Statistics Research Center (CRESS), U1153 Inserm, U1125 Inra, Cnam, Paris 13 University, 93000 Bobigny, France; 3 Public Health Departmant, Avicenne Hospital, Assistance Publique Hôpitaux de Paris (AP-HP), 93000 Bobigny, France

**Keywords:** front-of-pack nutrition label, traffic light, health star, Nutri-Score, reference intake, warning label

## Abstract

Consumers’ perceptions of five front-of-pack nutrition label formats (health star rating (HSR), multiple traffic lights (MTL), Nutri-Score, reference intakes (RI) and warning label) were assessed across 12 countries (Argentina, Australia, Bulgaria, Canada, Denmark, France, Germany, Mexico, Singapore, Spain, the UK and the USA). Perceptions assessed included liking, trust, comprehensibility, salience and desire for the label to be mandatory. A sample of 12,015 respondents completed an online survey in which they rated one of the five (randomly allocated) front-of-pack labels (FoPLs) along the perception dimensions described above. Respondents viewing the MTL provided the most favourable ratings. Perceptions of the other FoPLs were mixed or neutral. No meaningful or consistent patterns were observed in the interactions between country and FoPL type, indicating that culture was not a strong predictor of general perceptions. The overall ranking of the FoPLs differed somewhat from previous research assessing their objective performance in terms of enhancing understanding of product healthiness, in which the Nutri-Score was the clear front-runner. Respondents showed a strong preference for mandatory labelling, regardless of label condition, which is consistent with past research showing that the application of labels across all products leads to healthier choices.

## 1. Introduction

In response to rising rates of obesity around the world [1], front-of-pack labels (FoPLs) are increasingly being applied to pre-packaged foods to inform consumers about the nutritional value of these foods and help them to make healthier choices [2,3,4]. A large body of research supports the notion that FoPLs are more effective in achieving these aims compared to the provision of no nutrition information or just a nutrition facts panel (generally found on the back or side of packs) [5,6,7,8,9].

Of the different FoPL formats currently in use around the world, most can be classified as reductive or interpretive [4,10]. Reductive FoPLs provide factual information about a food (such as the amounts of key nutrients within a food) with minimal interpretation (such as the food’s contribution to an adult’s recommended daily intake). Interpretive FoPLs may contain similar information (i.e., amounts of key nutrients) but also use aids like colour to indicate the healthiness of the food. The reference intakes (RI) and multiple traffic lights (MTL) are prominently studied examples of reductive and interpretive labels respectively [8,9,11,12].

**Figure 1:**
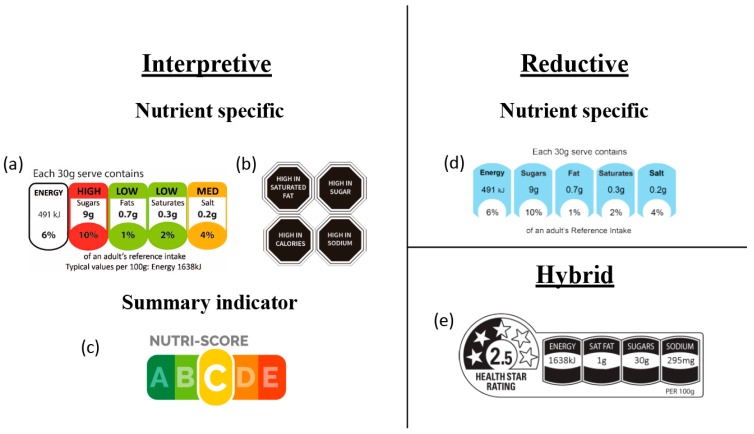
Examples of different front-of-pack label (FoPL) formats and their classifications: (**a**) multiple traffic lights; (**b**) warning labels; (**c**) Nutri-Score; (**d**) reference intakes; and (**e**) health star rating.

Interpretive FoPLs can further be divided into nutrient-specific or summary indicator formats. Interpretive nutrient-specific formats (such as the MTL) provide information on the individual nutrients within a food, while interpretive summary indicator formats provide an overall evaluation of the nutritional quality of the product. The warning label is an example of another interpretive nutrient-specific format that has recently been mandated in a number of countries [4]. This FoPL typically appears as a black hexagon with the text “High in” followed by saturated fat, salt, sugar, or calories when a predetermined threshold is exceeded. The Nutri-Score is an example of an interpretive summary indicator, assigning foods with a colour-coded rating from A to E. Finally, the health star rating (HSR) similarly features a summary indicator but also displays nutrient-specific information alongside the indicator, making it a hybrid FoPL. Visual depictions of these FoPLs can be found in Figure 1.

Studies have examined how the FoPLs described above influence people’s understanding of nutrition information and affect food choices [8,9,11,12]. Given the challenges of conducting research in supermarkets [13], these studies have typically been carried out online or within a laboratory setting [14]. The latter designs allow a high degree of control over the variables being assessed. However, it is possible that respondents are more motivated and less time-pressured to make healthy choices in these contexts. Asking consumers about how they perceive different labels (e.g., whether they like them, trust them, find them easy to use) could provide additional information on how likely shoppers are to actually use a given label. In addition, consumers’ attitudes towards FoPLs can affect whether or not governments choose to implement them [12,15]. One example of this comes from France, where consumers petitioned French retailers and food manufacturers to implement the Nutri-Score [15]. This eventually resulted in official recognition from the French government and uptake by some large retailers and manufacturers [15].

Past research on consumers’ perceptions of various FoPLs shows that people like simplified labels but want to know how the information underlying the label was derived and do not like to feel they are being coerced [12]. In past studies, consumers have reported positive attitudes towards the MTL [16,17,18,19,20,21,22,23,24,25], HSR [26], Nutri-Score [27,28] and RI [20,21,29,30,31]. However, a direct comparison of perceptions of these FoPLs (and warning labels) has not been performed to date.

FoPL comparison testing has global policy implications. In 2016, the International Association of Consumer Food Organizations proposed that the Codex Committee on Food Labelling develop a new global standard for interpretive FoPLs [32]. A unifying standard was described as having the ability to potentially “protect existing laws from World Trade Organization (WTO) challenge, and encourage and empower other countries to issue nutrition regulations with higher public health impact without fear of WTO disputes” (p. 2, [32]). If a common FoPL standard is to be used across many countries, it is of critical importance to investigate which FoPLs are most effective and well-received across many countries. Testing different FoPL formats provides information that can be considered when determining appropriate elements to include in global FoPL standards.

The FOP-ICE (Front-Of-Pack International Comparative Experiment) project was borne out of efforts to address this issue. Using a randomised experimental design, The FOP-ICE study assessed reactions to five different FoPLs (HSR, MTL, Nutri-Score, RI, warning label) among a large (*n* = 12,015), diverse sample of consumers from 12 countries (Argentina, Australia, Bulgaria, Canada, Denmark, France, Germany, Mexico, Singapore, Spain, the UK and the USA). Results on the relative effectiveness of the five FoPLs to enhance consumers’ understanding of the healthiness of food products showed that the Nutri-Score performed best across all countries, followed by the MTL [33]. The aim of the present study was to further interrogate the FOP-ICE study data by examining how respondents’ perceptions of FoPLs vary according to FoPL type and country of residence. Perceptions were assessed in terms of liking, trust, comprehensibility, salience and desire for the label to be compulsory. It was hypothesised that respondents would be most favourable to interpretive labels, as past research has shown that these are most useful in guiding consumers to healthier food choices [8,9,11,12].

## 2. Materials and Methods

Relevant information on the methodology of the present study is reported below. Further details on the broader FOP-ICE project can be found at http://www.ANZCTR.org.au/ACTRN12618001221246.aspx and elsewhere [33].

### 2.1. Participants

Respondents (*n* = 12,015) were recruited from 12 countries (Argentina, Australia, Bulgaria, Canada, Denmark, France, Germany, Mexico, Singapore, Spain, the UK and the USA) to participate in an online survey. All respondents gave their informed consent for inclusion before they participated in the study. The protocol of the present study was approved by the Curtin University Human Research Ethics Committee (approval reference: HRE2017-0760) and the Institutional Review Board of the French Institute for Health and Medical Research (IRB Inserm n_17-404). Recruitment was undertaken by an ISO-accredited international web panel provider (PureProfile). To ensure a diverse sample, quotas were applied so that the sample was evenly split according to gender, age (within the following brackets: 18–30 years, 31–50 years, >50 years) and income level (low, medium and high) within each country. Once a quota had been filled, panel members falling within that demographic group were not eligible to participate. Income brackets for each country were calculated around the median household income (based on various national statistical databases) for that country. A bracket of +/-33% was created around the median income to represent a ‘medium’ income band. Any incomes that fell below or above those figures were considered low or high, respectively. Key participant demographic data can be found in Table 1.

### 2.2. Procedure

The survey began with some background questions (e.g., gender, age, income, grocery buyer status, education level, nutrition knowledge and diet quality). Next, respondents were presented with three sets of three fictional food products with no FoPL on-pack. They were then randomised to one of the five FoPL conditions (HSR, MTL, Nutri-Score, RI, or warning label) and presented with the same food products (this time with a FoPL on-pack). In both the no-FoPL and FoPL scenarios, respondents were asked to (i) rank the three products within each set according to healthiness and (ii) select which product they would be most likely to buy (see [33] for results relating to healthiness rankings). At the conclusion of the study, respondents were presented with 9 items assessing their perception of the FoPL they had just seen. The items, which were assessed on a scale from 1 (strongly disagree) to 9 (strongly agree), were as follows:

- I like this label;
- I trust this label;
- This label is easy to understand;
- This label took too long to understand;
- This label is confusing;
- This label provides me with the information I need;
- This label does not stand out;
- It should be compulsory for this label to be shown on packaged food products;
- Food companies should be able to choose whether they apply this label to their packaged foods.

### 2.3. Analysis

Data analysis was performed in SPSS (version 25, SPSS Inc., Chicago, IL, USA). Given that some items were positively valanced and others were negatively valanced, respondents who provided the same response across all items (except those who responded with a 5, which was the mid-point of the scale) were removed from analyses (*n* = 203; 2% of the sample). This was a cautionary measure to eliminate potentially invalid responses. A 12 (country) × 5 (FoPL condition) ANCOVA was conducted on each perception item. The interaction between country and FoPL was also included as an independent variable. The *p*-value cut-off for significance (with a Bonferroni correction for 9 tests) was set to 0.005. Age, gender, income bracket, education, grocery buyer status, nutrition knowledge and diet were included as covariates. Post hoc comparisons among FoPLs and countries were performed with a Sidak correction for multiple comparisons applied to each survey item. The estimated marginal means for the different FoPLs (as well as the aggregated mean) and the FoPL by country interactions were graphed for the perception items where a significant main effect of FoPL or interaction between FoPL and country was observed, along with 99% confidence intervals to facilitate comparisons across all FoPLs.

## 3. Results

The mean score, standard deviation and intercorrelation for each perception item are shown in Table 2. Liking of and trust in a label were the most highly correlated items (*r* = 0.65). An unexpectedly low correlation (*r* = −0.16) was noted between the items assessing whether the label viewed by the respondent should be compulsory and whether food companies should be able to choose to apply the label. This may have been due to some respondents interpreting the latter item as asking whether food companies should have a choice to include the label vs. no label or interpreting it as asking whether companies should include the label vs. another label format. This item also showed the largest spread (SD = 2.78), indicating that there was less agreement among respondents on this item. Thus, no further analyses are reported on this item. The item assessing whether FoPLs should be compulsory on packs received the highest mean score (M = 7.13), indicating that respondents felt very strongly about this issue. In fact, 36.9% of the sample selected the highest score (9—‘strongly agree’) on this item.

**Figure 2:**
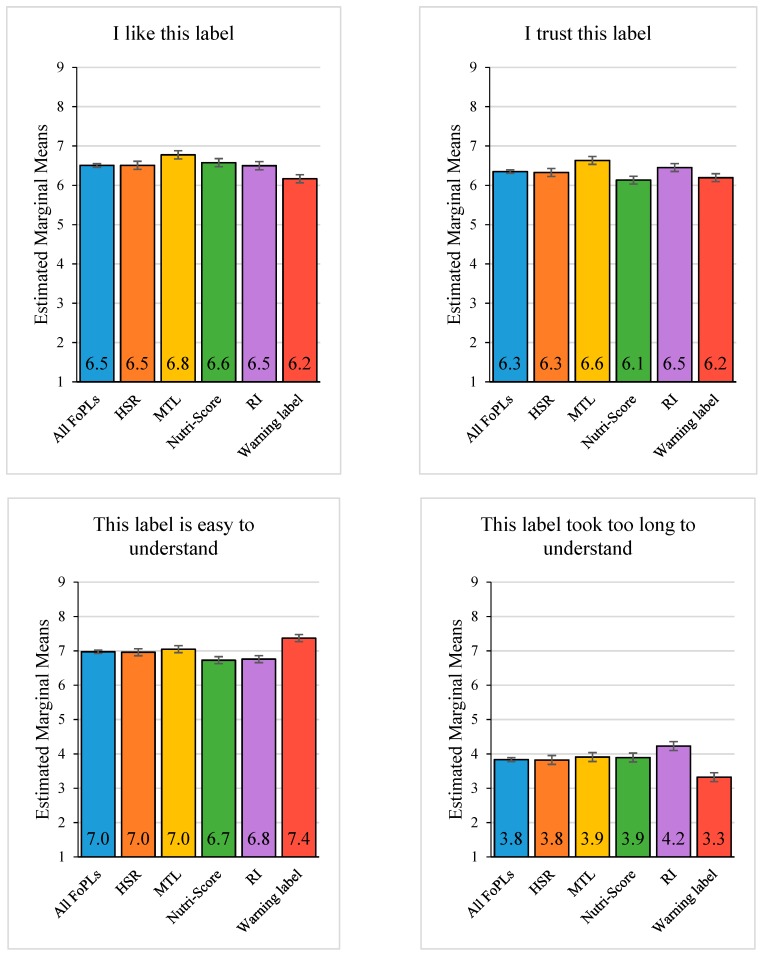

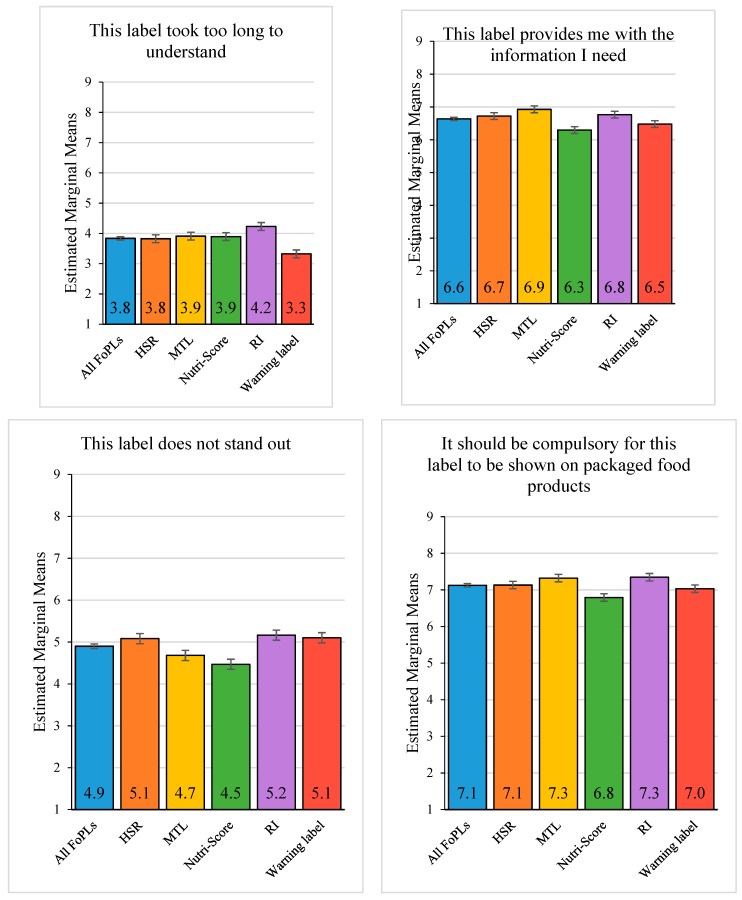
Mean scores across perception items for all FoPLs combined and individually. Note: Graphs show estimated marginal means for FoPL condition adjusted for age, gender, socioeconomic status, grocery buyer status, level of education, diet and nutrition knowledge. Error bars show 99% confidence intervals. HSR = Health Star Rating, MTL = Multiple Traffic Lights, RI = Reference Intakes.

**Figure 3:**
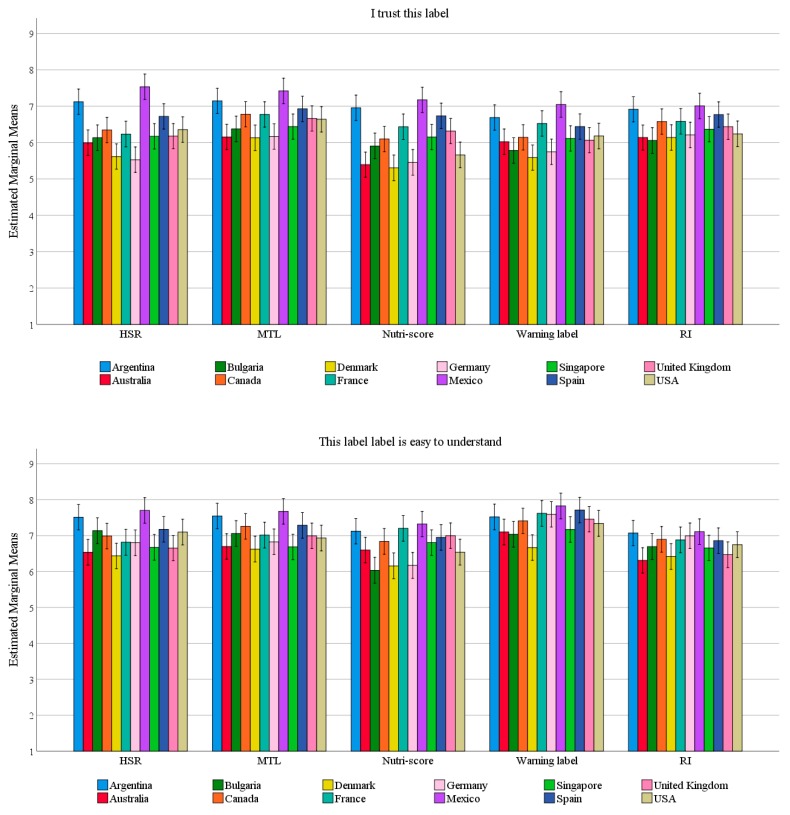

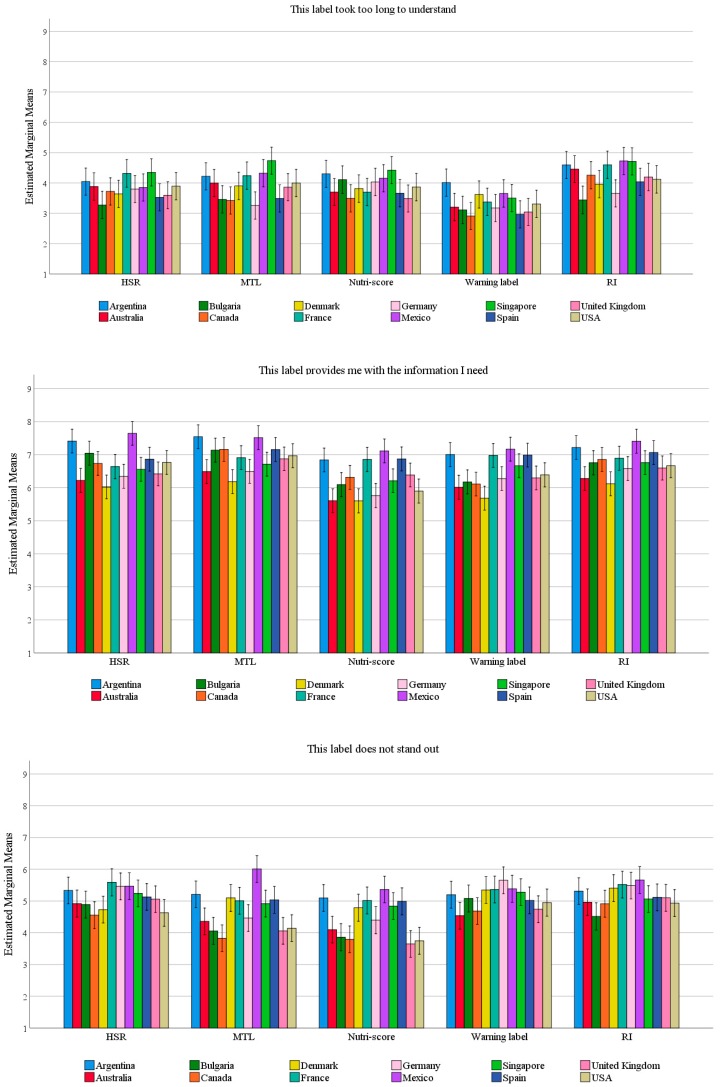

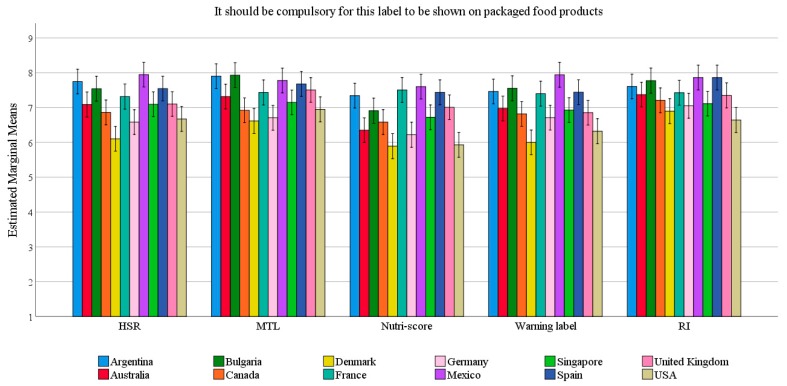
Mean scores across perception items according to country and FoPL type. Note: Graphs show estimated marginal means for countries adjusted for age, gender, SES, grocery buyer status, level of education, diet and nutrition knowledge. Error bars show 99% confidence intervals.

FoPL condition and country were significant predictors in the ANCOVAs across all 8 items (*p* < 0.0001). Graphs showing the distribution of means according to the different levels of these variables are presented in Figure 2 and Figure 3.

Some notable trends were observed among the FoPLs across the different perception items. Across all the FoPLs included in the present study, the MTL was perceived most favourably. It received the highest scores out of all the FoPLs on four criteria (trust, liking, ease of understanding and providing needed information), the second highest score on the item assessing whether the FoPL should be compulsory and the second lowest score on the item “This label does not stand out”. Respondents were ambivalent about the RI and Nutri-Score and neutral about the warning label and HSR. The RI received the highest mean scores for being confusing and not standing out, although it was relatively well trusted and perceived to be appropriate as a compulsory FoPL. The Nutri-Score received the lowest mean scores on trust, being easy to understand, providing enough information and being appropriate as a compulsory label on food packs, but it was relatively well liked and was perceived as standing out more than the other FoPLs. The warning label was the easiest label to interpret (scoring highest on ease of understanding and lowest on being confusing and taking too long to understand) but received the lowest score for liking. Perceptions of the warning label in relation to the other criteria tended to lie somewhere between those of the other FoPLs. The HSR received a relatively high score on the “does not stand out” item and fell somewhere between the other FoPLs for all the other items.

The country x FoPL interaction was significant (*p* < 0.005) for 6 of the 8 perception items: “I trust this label”, “This label is easy to understand”, “This label took too long to understand”, “This label provides me with the information I need”, “This label does not stand out” and “It should be compulsory for this label to be shown on packaged food products”. Graphs showing the interaction for these items can be seen in Figure 3.

Looking across the interactions, no consistent trends were observed across the items. Patterns of differences between countries were not the same across different FoPL conditions or different perception items.

## 4. Discussion

This study explored consumers’ perceptions of five FoPLs that are currently used around the world. The results show that irrespective of how favourably the different FoPLs were perceived or the country of residence, there was a clear demand for front-of-pack nutrition information to be made available. This was demonstrated in the very high mean score (7 out of 9) and a third of the sample selecting 9 (‘strongly agree’) for the item assessing whether the FoPL to which they were exposed should be compulsory on packs. Previous research supports the need for mandatory FoPLs, with supermarket studies reporting increased sales of healthier foods when FoPLs are applied to all products within a category rather than just a selection of products [34,35,36,37]. Although FoPLs should aid consumers in assessing the healthiness of individual products in isolation, they are most useful when they also allow consumers to compare healthiness across multiple products [38]. Some FoPLs, such as the warning label, only work if they are compulsory given that food manufacturers have no incentive to apply a FoPL across their product range when the aim of the FoPL is to reduce purchases of a product. Furthermore, past experience with the HSR has shown that when FoPLs are not mandatory, they skew towards appearing on healthier products [39], and this can reduce consumer trust in the system as a whole [39,40].

Looking at perceptions of the individual FoPLs studied, it is evident that the MTL was most favourably perceived. Respondents liked and trusted this FoPL the most and felt it provided the information they needed and was the easiest to understand. Perceptions of the RI and Nutri-Score were mixed. Respondents reported that the RI stood out the least and was the most confusing, but they showed relatively high trust in it and felt it should be compulsory on packs. Conversely, the Nutri-Score reportedly stood out the most and was easiest to understand but was the least trusted and least desired as a compulsory FoPL. Although the warning label was considered easiest to interpret, it was least liked. This may be due to the stark negative nature of this label. Finally, the HSR was perceived to stand out the least, which may go some way towards explaining why this FoPL tended to fall somewhere in between the other FoPLs on the other perception dimensions. It is important to note that the absolute differences between the FoPLs tended to be small (i.e., rarely more than 0.5 points difference on the 5-point scale), and thus, in some cases, it is more informative to consider the rating that was averaged across all FoPLs. These findings suggest that, on the whole, respondents were favourable towards FoPLs in general.

Looking at trends among FoPLs that share similar features, it is clear that the coloured FoPLs (MTL and Nutri-Score) stood out the most and were most liked. The more simplified FoPLs (Nutri-Score and warning labels) were seen as not providing enough information and were least trusted and less likely to be desired as compulsory. Other findings from this dataset found the Nutri-Score to be most useful in assisting consumers to accurately identify the healthiest food from a choice set [33]. This is discrepant with the present results showing that this FoPL was perceived to not provide enough information and to be harder to understand. These results suggest that consumers could benefit from education on the credibility of highly interpretive FoPLs such as the Nutri-Score to foster trust in the system, motivate consumers to make use of it and bring perceptions in line with performance.

Respondents were most in favour of the MTL and RI being compulsory on food packs. This is interesting given that other results from this same dataset show that these two FoPLs produce opposite outcomes on objective understanding [33]. Specifically, the MTL led to more positive outcomes (after the Nutri-Score) while the RI performed most poorly. These results are in line with previous studies demonstrating that consumers perceive that more information is better [41]. However, most consumers are not equipped to interpret all this information due to factors such as low levels of nutrition knowledge [42], time pressure [12] and competing priorities [43]. This is evident when results from the perception elements assessed in the present study (which show that consumers desire more information) are compared to the objective understanding results [33] (which show that understanding of food healthiness is not always improved by more information). Fortunately, respondents’ perceptions were not always discrepant with objective understanding, as was the case with the MTL, which performed relatively well across both objective understanding and consumer preference. Although some differences were noted between countries, no meaningful or consistent patterns were present in the interactions between country and FoPL type.

It is important to note that certain elements of the study design are likely to have influenced the results. First, the between-subjects design meant that respondents provided ratings for only one FoPL. If respondents had been asked to rank the FoPLs, a clearer hierarchy may have emerged. However, only one FoPL was shown in order to keep the experimental tasks (completed before the perception ratings) to a management time limit. That differences were found among FoPLs using a design that is less sensitive for detecting differences is notable. Second, respondents were asked to provide their opinions directly after using a specific FoPL to make decisions about food healthiness and choice. This means that FoPL perceptions were grounded in the first-hand experience of respondents, which increases the ecological validity of the findings. However, one limitation is that the experimental process did not permit replication of any tactile experiences that would be available to customers in real-world supermarkets.

## 5. Conclusions

Overall, the results suggest that interpretive aids such as colour are viewed favourably by consumers but oversimplified FoPL formats risk excluding information that is desired by consumers and as a consequence being less trusted. Across the large and diverse sample of respondents, there was strong demand for FoPLs to be compulsory on food packs. This is an important message for policy makers to take away from these findings and is consistent with results from previous studies showing that FoPLs are most effective when applied to all products within a choice set, thus facilitating product comparisons and reducing the cognitive load on shoppers. Perceptions are just one dimension on which consumers’ reactions to FoPLs can be assessed. Future work should consider how food choices in the real world are affected across culturally diverse groups.

## Figures and Tables

**Table 1:** Key respondent demographic information.

	All Countries		Argentina		Australia		Bulgaria		Canada		Denmark		France		Germany		Mexico		Singapore		Spain		UK		USA	
*n*	11812		992		987		987		984		978		977		985		987		989		984		990		972	
	n %		n %		n %		n %		n %		n %		n %		n %		n %		n %		n %		n %		n %	
Gender																										
Males	5889	50	488	49	492	50	490	50	490	50	492	50	485	50	493	50	495	50	495	50	495	50	493	50	481	49
Females	5923	50	504	51	495	50	497	50	494	50	486	50	492	50	492	50	492	50	494	50	489	50	497	50	491	51
Age, Years																										
18–30	3951	33	329	33	323	33	348	35	332	34	316	32	326	33	334	34	335	34	333	34	332	34	327	33	316	33
31–50	3969	34	330	33	332	34	366	37	323	33	326	33	323	33	326	33	326	33	335	34	325	33	332	34	325	33
>50 years	3892	33	333	34	332	34	273	28	329	33	336	34	328	34	325	33	326	33	321	32	327	33	331	33	331	34
Level of Income																										
Low	3896	33	331	33	321	33	273	28	334	34	329	34	324	33	333	34	333	34	335	34	331	34	323	33	329	34
Medium	3985	34	331	33	332	34	350	35	329	33	336	34	329	34	329	33	325	33	334	34	326	33	334	34	330	34
High	3931	33	330	33	334	34	364	37	321	33	313	32	324	33	323	33	329	33	320	32	327	33	333	34	313	32

**Table 2:** Means, standard deviations and Pearson correlation coefficients between the perception items.

	Mean	Standard Deviation	I Like This Label	I Trust This label	This Label is Easy to Understand	This Label Took Too Long to Understand	This Label is Confusing	This Label Does Not Stand Out	This Label Provides Me with the Information I Need
I like this label+	6.5	2.0							
I trust this label+	6.3	2.0	0.65						
This label is easy to understand+	7.0	2.0	0.58	0.54					
This label took too long to understand−	3.8	2.5	−0.20	−0.15	−0.43				
This label is confusing−	3.7	2.4	−0.29	−0.24	−0.47	0.70			
This label does not stand out−	4.9	2.4	−0.13	−0.06	−0.12	0.39	0.38		
This label provides me with the information I need+	6.6	2.0	0.64	0.64	0.59	−0.22	−0.31	−0.08	
It should be compulsory for this label to be shown on packaged food products+	7.1	2.0	0.52	0.49	0.48	−0.19	−0.26	−0.02^a^	0.55

^+^ Positively valanced item. − Negatively valanced item ^a^ *p* = 0.04. All other correlations significant at *p* < 0.005.
